# Wild Hedgehogs and Their Parasitic Ticks Coinfected with Multiple Tick-Borne Pathogens in Jiangsu Province, Eastern China

**DOI:** 10.1128/spectrum.02138-22

**Published:** 2022-08-24

**Authors:** Yong Qi, Lele Ai, Changqiang Zhu, Fuqiang Ye, Ruichen Lv, Junhu Wang, Yingqing Mao, Nianhong Lu, Weilong Tan

**Affiliations:** a Huadong Research Institute for Medicine and Biotechniques, Jiangsu, China; b Nanjing Bioengineering (Gene) Technology Center for Medicines, Jiangsu, China; c Institute of Rocket Force Medicine, State Key Laboratory of Trauma, Burns and Combined Injury, Army Medical University, Chongqing, China; Changchun Veterinary Research Institute

**Keywords:** *Haemaphysalis flava*, coinfection, tick, hedgehog, *Rickettsia*, mNGS, tick-borne pathogen

## Abstract

The increasing awareness of emerging tickborne pathogens (TBPs) has inspired much research. In the present study, the coinfections of TBPs both in ticks and their wild hedgehog hosts in Jiangsu province, Eastern China were determined by metagenome next-generation sequencing and nested PCR. As a result, Rickettsia japonica (81.1%), novel *Rickettsia* sp. SFGR-1 (5.1%), Anaplasma bovis (12%), A. platys (6.3%), novel *Ehrlichia* spp. Ehr-1 (16%) and Ehr-2 (0.6%), E. ewingii-like strain (0.6%), Coxiella burnetii (10.9%), and a novel *Coxiella*-like endosymbiont (CLE) strain (61.1%) were detected in *Haemaphysalis flava* ticks. A. bovis (43.8%), *Ehrlichia* sp. Ehr-1 (83.3%), and C. burnetii (80%) were detected in Erinaceus amurensis hedgehogs. Coinfection rates with various TBPs were 71.5% and 83.3% in ticks and hedgehogs, respectively, both with double-pathogen/endosymbiont coinfection rates over 50%. We found the following. (i) *Er. amurensis* hedgehogs seem to contribute to the natural cycles of *R. japonica*, *A. bovis*, *Ehrlichia* sp., and C. burnetii and may be reservoirs of them except for *R. japonica*, and *A. bovis* is proved to infect hedgehogs for the first time. (ii) *H. flava* is proved to harbor various TBPs as a reservoir host, including CLE identified for the first time, which could inhibit coinfection of C. burnetii while promoting that of *Rickettsia* spp. in *H. flava.* (iii) Four novel TBP species were identified. This study provides useful epidemiological information crucial for assessing the potential infection risks to humans, thus benefiting the development of strategies to prevent and control tick-borne diseases.

**IMPORTANCE** In the present study, we found the following. (i) *Er. amurensis* hedgehogs seem to contribute to the natural cycles of *R. japonica*, *A. bovis*, *Ehrlichia* sp., and C. burnetii and may be reservoirs of them except for *R. japonica*, and *A. bovis* is proved to infect hedgehogs for the first time. (ii) *H. flava* is proved to harbor various tickborne pathogens (TBPs) as a reservoir host, including *Coxiella*-like endosymbiont (CLE) identified for the first time, which could inhibit coinfection of C. burnetii while promoting that of *Rickettsia* spp. in *H. flava.* (iii) Four novel TBP species were identified. This study provides useful epidemiological information on TBPs harbored and transmitted by ticks and their hosts, for assessing the potential infection risks to humans, thus benefiting the developing strategies for tick-borne diseases prevention and control.

## INTRODUCTION

Ticks transmit various pathogens, such as bacteria, viruses, protozoa, and helminths, causing serious diseases or even death in both animals and humans (1 to 3). In light of the ongoing geographical expansion of ticks caused by climatic change, as well as their increased ability to harbor new pathogens, public health concerns have been raised for both humans and animals (4, 5). During 1950 to 2018, China reported 103 tick-borne agents, including 29 species (subspecies) of pathogens, with most discovered in the last decades (5), indicating the serious public health threat that the tickborne diseases have imposed in China.

Hosts of ticks include domestic animals, wild animals, and humans; wild hedgehogs tend to be highly infested by ticks and highly at risk for tick-borne diseases (6, 7). In urban or suburban areas, hedgehogs are thought to play a significant role in the dissemination and transmission of various tick-borne pathogens (TBPs) such as Borrelia spielmanii, B. bavariensis, B. afzelii, Rickettsia helvetica, and Anaplasma phagocytophilum as well as their enzootic cycles (8). Various pathogens have different geographical distributions, and the role of hedgehogs and their parasitic ticks in tick-borne diseases is still not fully understood (6). Hedgehogs' ecological habits dictate their close relationship with humans. Also, nowadays, with an expanding range of human activities, there is a much greater chance of exposure of humans to hedgehogs and their harboring ticks. Consequently, studies focusing on this topic should be undertaken (8).

The present study tested for the existence and coexistence of TBPs within *Haemaphysalis flava* ticks and their hedgehog hosts collected in Jiangsu province, Eastern China, by combining metagenomic next-generation sequencing (mNGS) and nested PCR. We hoped the findings could provide a deeper epidemiological understanding of the pathogens harbored by ticks and their hedgehog hosts as well as valuable data for evaluating the potential risk of tick-borne diseases from contact with wild hedgehogs or their contaminated environment, thus benefiting the prevention and control of the potential tick-borne diseases.

## RESULTS

### Taxonomic classification.

According to the partial mitochondrial large subunit rRNA gene sequencing and alignment (GenBank: ON016529), all the obtained hedgehogs were classified as *Erinaceus amurensis*. Six (3.3%) of the 181 adult ticks feeding on hedgehogs were identified to be Haemaphysalis longicornis, and the other 175 (96.7%) were identified as *H. flava* (GenBank: ON016525 to ON016528). Only *H. flava* was analyzed in this study due to the small sample sizes of H. longicornis. Female *H. flava* accounted for 74.9% (131/175) and male ones accounted for 25.1% (44/175).

**FIG 1:**
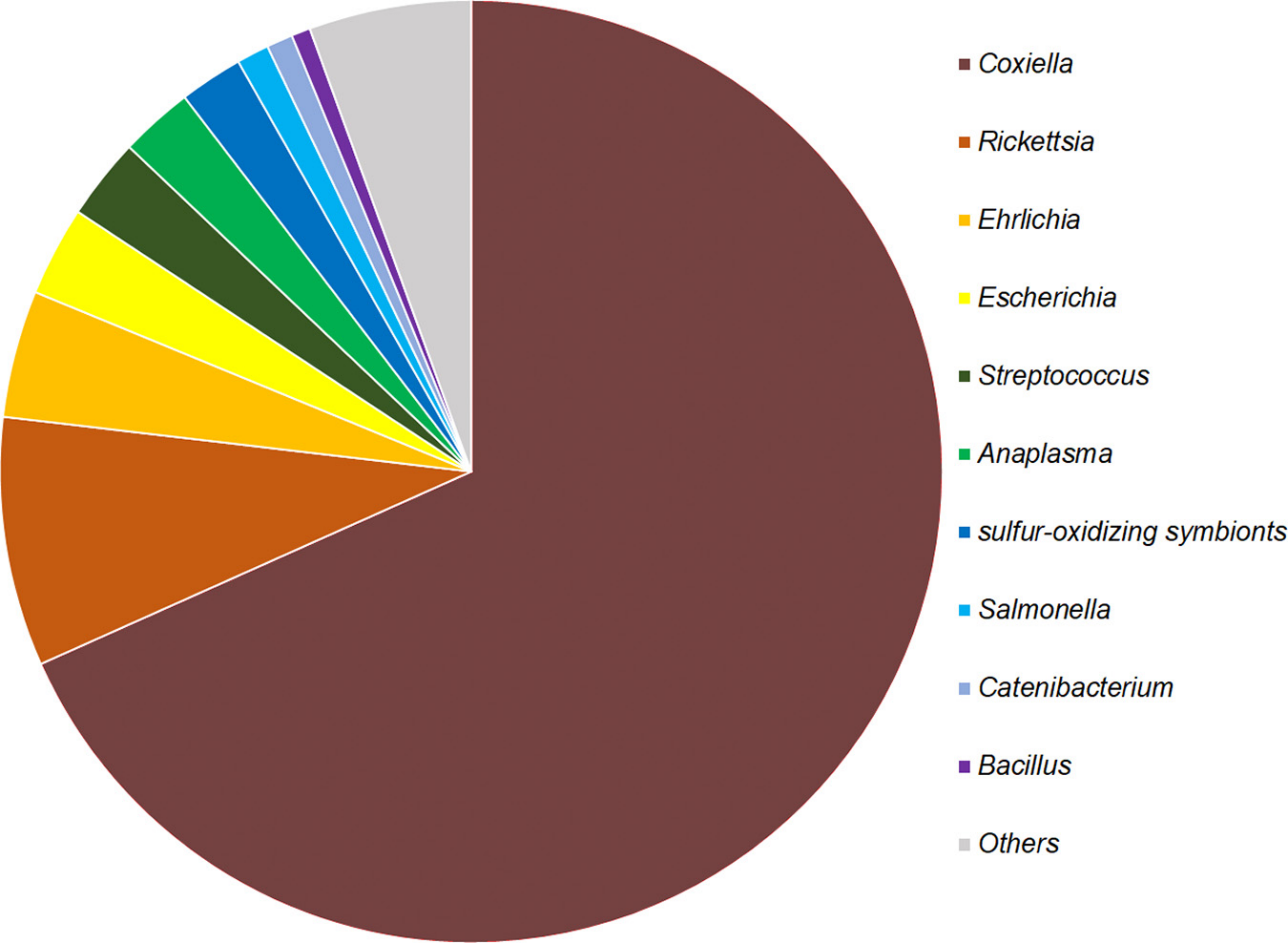
Relative abundances of potential top 10 pathogens at the genus level in the pooled *H. flava* sample analyzed by metagenomic next-generation sequencing.

In mNGS of the pooled DNA samples, sequencing yielded a total of 90 million reads and 13.5 billon bases with GC bases ratio of 48.32% and high quality of Q20 = 95.28%, which rose to 97.20% after treatment with Trimmomatic. The relative abundance was measured at various taxonomic levels (Fig. S2). Briefly, 11.2% of the obtained open reading frames (ORFs) belonged to microorganisms, including bacteria (10.98%), archaea (0.04%), viruses (0.05%), and fungi (0.13%). Taxonomic profiles at the genus level confirmed that genera *Rickettsia*, *Anaplasma*, *Ehrlichia*, and *Coxiella* existed in the pooled sample, which all predominated the top 6 in relative abundance (Fig. 1 and Fig. S2). Genus *Coxiella* was most abundant in the pool.

### Prevalence of TBPs in ticks and hedgehogs.

**FIG 2:**
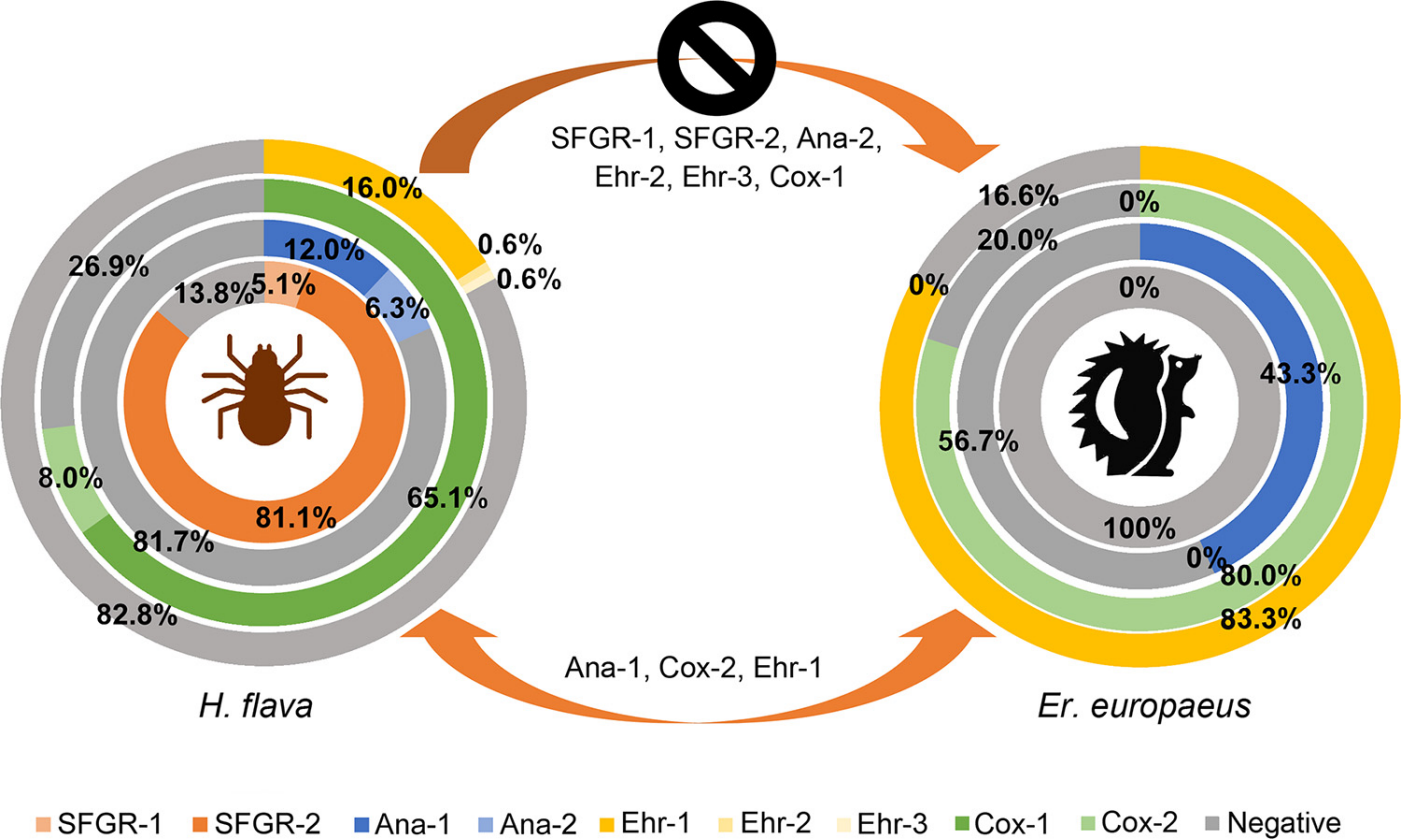
The observed positive rates and potential mutual communications of various pathogens in the *H. flava* ticks (*n* = 175) and *Er. amurensis* hedgehogs (*n* = 30). The identified *Rickettsia* spp. SFGR-1 and SFGR-2, *Anaplasma* spp. Ana-1 and Ana-2, *Ehrlichia* spp. Ehr-1, Ehr-2, and Ehr-3, and *Coxiella* spp. Cox-1 and Cox-2 in this study were indicated. The black forbidden symbol indicates the pathogens in *H. flava* didn’t spread to or establish infection in hedgehogs.

Nested PCR was performed on each sample of *H. flava* to confirm *Rickettsia*, *Anaplasma*, *Ehrlichia*, and *Coxiella*. Representative results of agarose gel electrophoresis analysis of the amplified products of nested PCR toward partial *rrs* genes of *Anaplasma* spp. and *Ehrlichia* spp are shown in Fig. S3. As a result (Fig. 2), in *H. flava* ticks from hedgehogs, 86.3% (151/175) were positive for *Rickettsia*, 15.4% (27/175) positive for *Anaplasma*, 17.1% (30/175) positive for *Ehrlichia*, and 71.4% (125/175) positive for *Coxiella*. Overall, in the 175 H. flava ticks, only 6 were negative for any of the pathogens of the 4 genera of pathogens.

**FIG 3:**
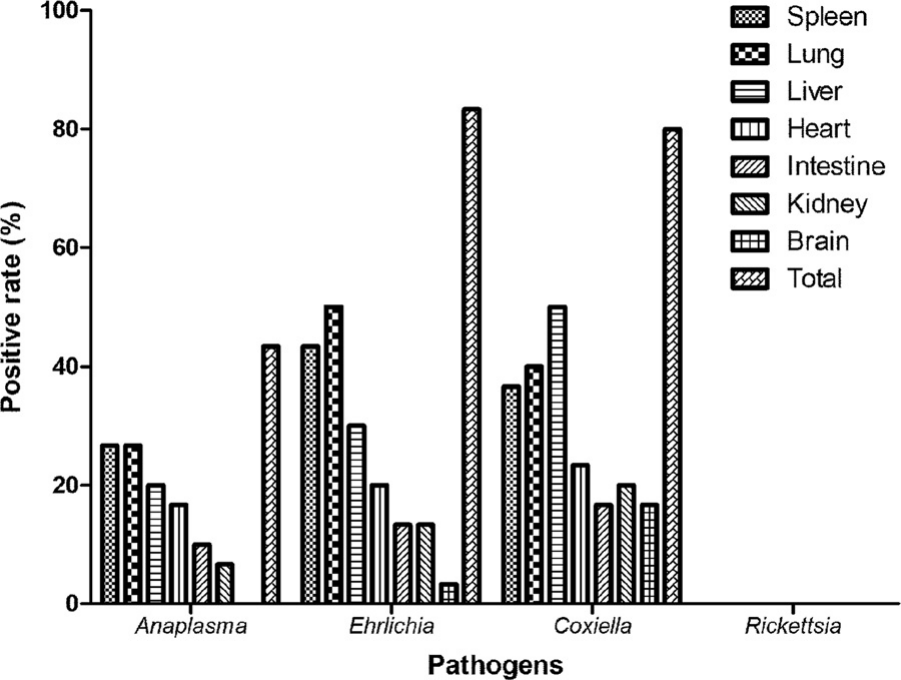
Positive rates of the tick-borne pathogens in different organs of the hedgehogs (*n* = 30).

In hedgehogs, different organs performed different positive rates for the TBPs (Fig. 3). In total, spleens, lungs, and livers represented the top 3 organs harboring *Anaplasma*, *Ehrlichia*, and *Coxiella* with the highest positive rates. The total positive rates of *Anaplasma*, *Ehrlichia*, and *Coxiella* in the hedgehogs were 43.8% (13/30), 83.3% (25/30), and 80% (24/30), respectively. To our surprise, the *rrs* gene of *Rickettsia* was not detected in any organs of the hedgehogs. Another set of primers targeting the *gltA* gene of spotted fever group *Rickettsia* (SFGR) was further used for nested PCR amplification of the samples from these hedgehogs, and the same result was observed, confirming the absence of *Rickettsia* in the hedgehogs. Overall, in the 30 hedgehogs, only 2 were negative for all of the 4 kinds of pathogens.

### Coinfection in individual ticks and hedgehogs.

**FIG 4:**
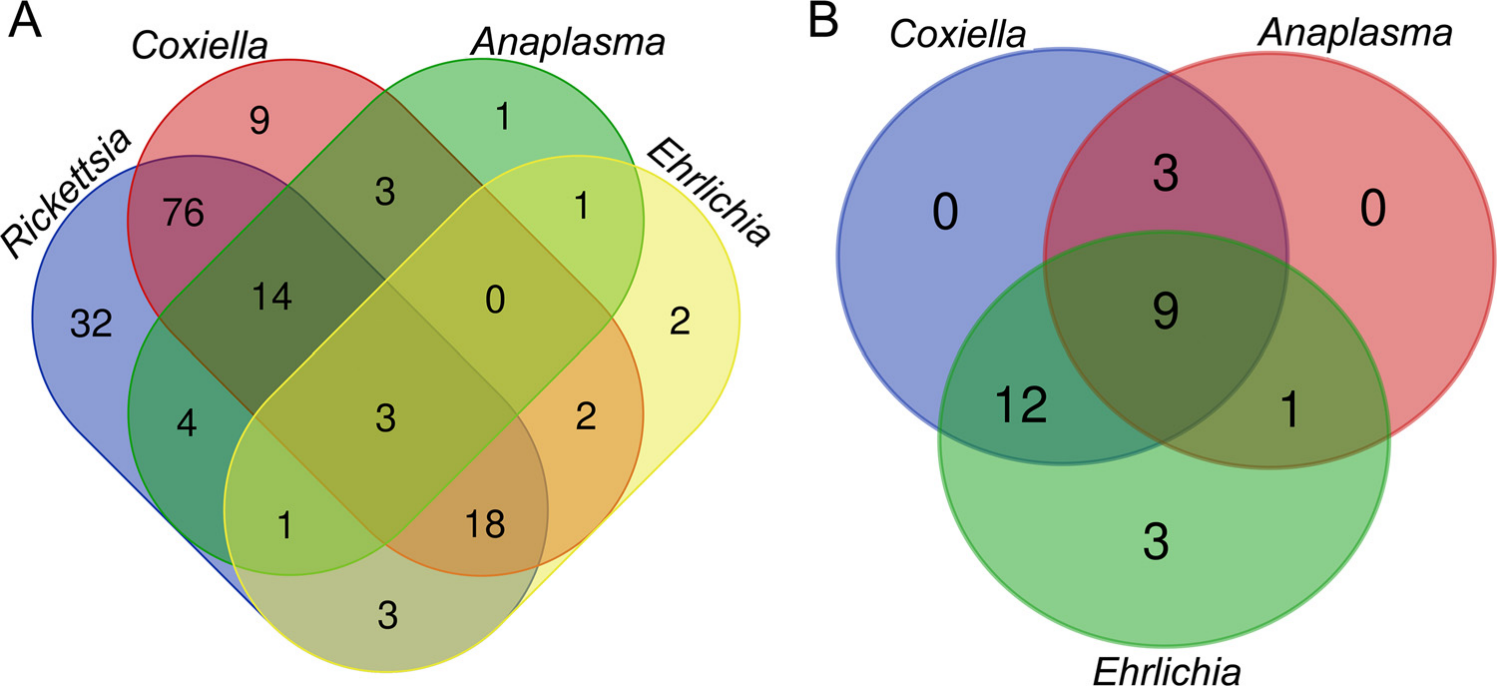
Coexistences of various pathogens in *H. flava* ticks (A) and their *Er. amurensis* hedgehog hosts (B) at genus level. Pathogens and numbers of individuals harboring various pathogens are indicated.

As shown in Table 1 and Fig. 4, 74.9% (131/175) of the *H. flava* ticks were coinfected with more than one pathogen identified in this study. The dual coinfection with *Coxiella* spp. and *Rickettsia* spp. was identified as predominantAs shown in (43.4%), and the triple coinfection with *Coxiella* spp., *Ehrlichia* spp., and *Rickettsia* spp. was also frequent. The quadra coinfection with all 4 species of pathogens was observed, though with a low rate of 1.7%.

**TABLE 1:** Infection and coinfection with tick-borne pathogens at genus level in individual *H. flava* ticks and *Er. amurensis* hedgehogs

Pathogens (genus)	No. of positive individuals	
	Ticks (*n* = 175)	Hedgehogs (*n* = 30)
Quadruple	3 (1.7%)	
*Anaplasma*, *Coxiella*, *Ehrlichia*, *Rickettsia*	3 (1.7%)	
Triple	33 (18.9%)	9 (30%)
*Anaplasma*, *Coxiella*, *Rickettsia*	14 (8%)	
*Coxiella*, *Ehrlichia*, *Rickettsia*	18 (10.3%)	
*Anaplasma*, *Ehrlichia*, *Rickettsia*	1 (0.6%)	
*Anaplasma*, *Ehrlichia*, *Coxiella*		9 (30%)
Double	89 (50.9%)	14 (53.3%)
*Coxiella*, *Rickettsia*	76 (43.4%)	
*Anaplasma*, *Rickettsia*	4 (2.3%)	
*Ehrlichia*, *Rickettsia*	3 (1.7%)	
*Anaplasma*, *Coxiella*	3 (1.7%)	3 (10%)
*Coxiella*, *Ehrlichia*	2 (1.1%)	12 (40%)
*Anaplasma*, *Ehrlichia*	1 (0.6%)	1 (3.3%)
Single	44 (25.1%)	3 (10%)
*Rickettsia*	32 (18.3%)	
*Coxiella*	9 (5.1%)	
*Anaplasma*	1 (0.6%)	
*Ehrlichia*	2 (1.1%)	3 (10%)
None	6 (3.4%)	2 (6.7%)

In hedgehogs, the dual pathogen coinfections with *Coxiella* spp. and *Ehrlichia* spp. and triple pathogen coinfections with *Anaplasma* spp., *Coxiella* spp., and *Ehrlichia* spp. took the lead with rates of 40% and 30%, respectively. The quadra coinfection was not observed due to the absence of *Rickettsia* spp. in hedgehogs.

Additionally, one species of each of the three genera, *Anaplasma*, *Ehrlichia*, and *Coxiella*, was found to infect both ticks and hedgehogs (Fig. 2).

### Phylogenetic analysis.

**FIG 5:**
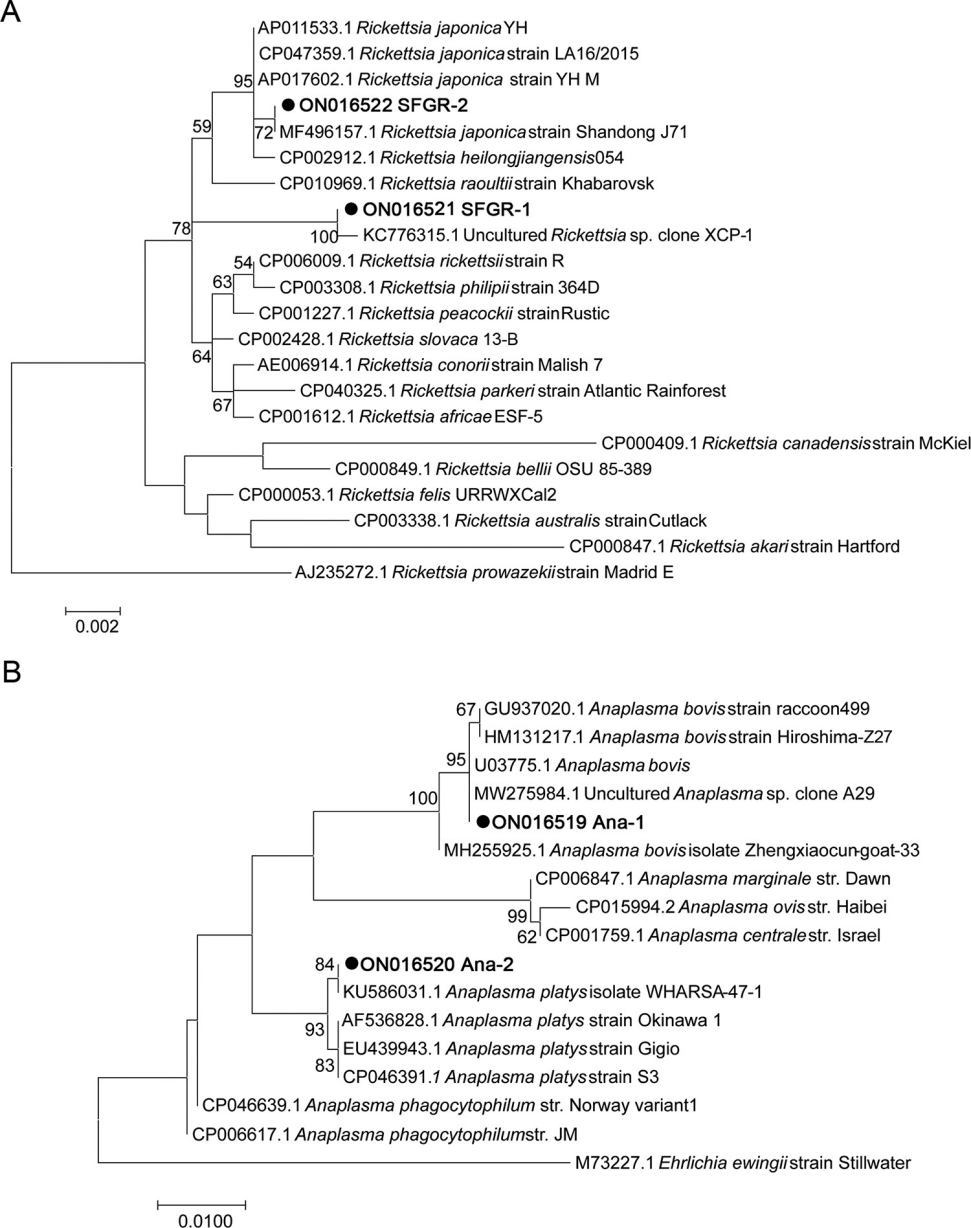
Phylogenetic analysis based on the partial *rrs* gene sequence of *Rickettsia* (A) and *Anaplasma* (B) species using MEGA 7.0 software. Maximum likelihood method with 1,000 replicates was done to generate the tree. A bootstrap value of greater than 50% was indicated. Nucleotide substitutions per site are indicated by the scale bar. The reference species and GenBank accession number of the sequence are shown on each line.

Two different partial *rrs* gene sequences of SFGR (SFGR-1 and SFGR-2 as indicated in Fig. 5A, GenBank: ON016521 and ON016522) were obtained from tick samples by nested PCR, indicating that two different SFGR strains existed in the samples. The sequence SFGR-1, amplified from 9 H. flava ticks, performed the highest homology (99.92% identity rate) with an uncultured *Rickettsia* sp. sequence (GenBank: KC776315.1) formerly identified in *H. longicornis* ticks in Beijing, China (9). They formed a separate branch in the phylogenetic tree (Fig. 5A), indicating they belonged to an unidentified novel species. The sequence SFGR-2, which was amplified from 142 ticks, performed the highest homology with the partial sequence from *R. japonica* strain Shandong J71 (99.71% identity rate) (GenBank: MF496157.1) identified in Shandong province, Northern China. Phylogenic analysis indicated that SFGR-2 was a strain of *R. japonica*, because it clustered with *R. japonica* and *R. heilongjiangensis* but was more closely related to *R. japonica* (Fig. 5A).

For identification of *Anaplasma* spp., a longer *rrs* gene was amplified, and two different sequences were obtained (GenBank: ON016519 and ON016520). Ana-1, performing 100% identity rate with the partial *rrs* gene sequence of an uncultured *Anaplasma* sp. clone A29 (GenBank: MW275984.1) found in *H. flava* in China as well as an undefined *A. bovis* strain found in South Africa (GenBank: U03775.1), formed a cluster with various *A. bovis* strains in phylogenic analysis (Fig. 5B). This *A. bovis* sequence was amplified in 21 ticks and 13 hedgehogs. However, Ana-2, performing 100% identity rate with the partial *rrs* gene sequence of an isolate of A. platys (GenBank: KU586031.1), formed a cluster with various strains of this species as well. It was only detected in 11 ticks but none of the hedgehogs. Interestingly, some tick samples were found to contain both kinds of sequences judged by overlapping peaks in the sequencing map, indicating coinfection of various *Anaplasma* species existed in *H. flava* ticks (data not shown).

**FIG 6:**
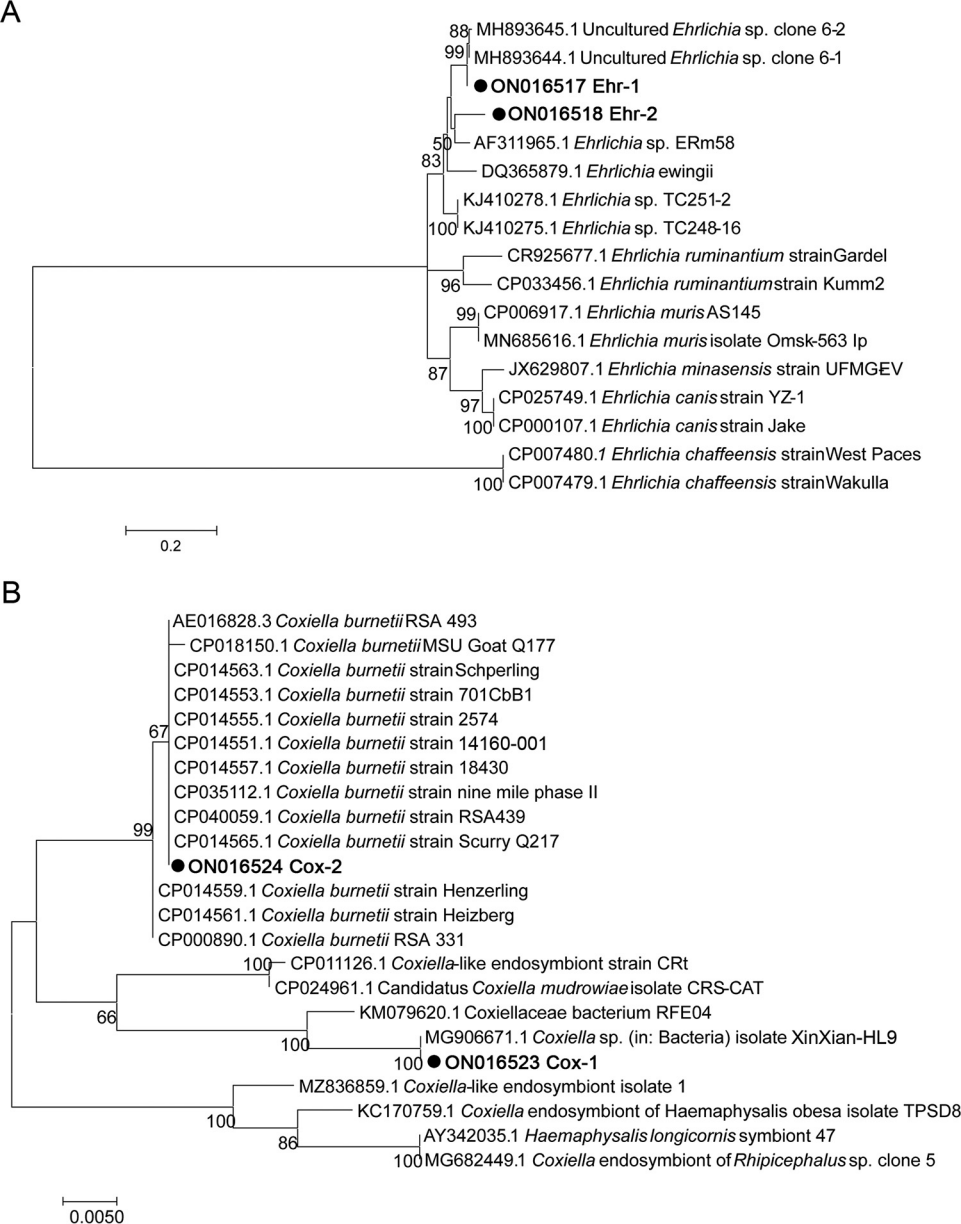
Phylogenetic analysis based on the partial gene sequences of *gltA* of *Ehrlichia* species (A) and *rrs* of *Coxiella* species (B) using MEGA 7.0 software. Maximum likelihood method with 1,000 replicates was done to generate the tree. A bootstrap value of greater than 50% is indicated. Nucleotide substitutions per site are indicated by the scale bar. The reference species and GenBank accession number of the sequence are shown on each line.

For *Ehrlichia* spp. identification, three different short sequences of the *rrs* gene were amplified from ticks (GenBank: ON016514 to ON016516), while only two partial *gltA* gene sequences were successfully amplified (GenBank: ON016517 and ON014518). Sequence of Ehr-1 performed 99.26% and 98.77% identity rates with partial sequences of *gltA* gene of two uncultured *Ehrlichia* species (GenBank: MH893644.1 and MH893645.1) hosted in *Er. amurensis*, respectively. They formed a cluster in the phylogenic tree (Fig. 6A). Similarly, Ehr-2 performed 91.11% identity rate with an undefined *Ehrlichia* sp. Erm58 (GenBank: AF311965.1) reported to belong to the *E. canis* group (10), which was identified in *Rhipicephalus muhsamae* ticks in African. Nevertheless, both Ehr-1 and Ehr-2 were believed to belong to novel *Ehrlichia* species. Though the *gltA* gene of the third *Ehrlichia* sp. was not amplified, the *rrs* sequence of this strain performed 99.59% identity rate with various strains or isolates of *E. ewingii* in the alignment analysis by BLAST (data not shown). In all, Ehr-1, Ehr-2, and the *E. ewingii*-like strain were identified in 28, 1, and 1 ticks, respectively, while only Ehr-1 existed in the hedgehogs with a high positive rate of 83.3% (25/30).

In phylogenic analysis of the obtained two partial sequences of the *rrs* gene (GenBank: ON016523 and ON016524) of *Coxiella* spp., Cox-1, performing high homologies of 99.44% and 99.31% with the sequences of two uncultured and undefined *Coxiella* spp. (GenBank: MG906671.1 and KC776319.1), respectively, and forming a separate branch together with “*Candidatus* C. mudrowiae isolate” (GenBank: CP011126.1) and *Coxiella*-like endosymbiont strain CRt (GenBank: CP024961.1), was difficult to be determined a novel *C. burntii* strain or an endosymbiont strain, while Cox-2 was believed to be a *C. burntii* strain by forming a cluster with various other *C. burntii* strains (Fig. 6B). Cox-1 was found in 114 ticks and none of the hedgehogs, while Cox-2 was found in 14 ticks and 24 hedgehogs. Also, 3 tick samples were found to contain both kinds of *Coxiella* spp., judged by overlapping peaks in the sequencing maps of *Anaplasma* species.

### Mutual effects of various pathogens.

Whether one pathogen/endosymbiont infection could promote or inhibit the other’s coinfection in ticks or hedgehogs was statistically analyzed. In the *H. flava* ticks, the *Coxiella* sp. (Cox-1) was observed to have the ability to significantly inhibit the coinfection of C. burnetii (continuity adjusted χ^2^ = 10.8, *P = *0.00102) and promote the coinfection of *Rickettsia* spp. (χ^2^ = 4.57, *P = *0.0326). However, mutual effects between other pathogens were not observed either in the ticks or hedgehogs (*P > *0.05).

## DISCUSSION

In recent years, the increasing awareness of emerging TBPs has greatly inspired studies on ticks and tick-borne diseases in China (11). Epidemiological information on ticks and their hosts, and their harboring and transmitting pathogens, can be useful for the development of tick-borne diseases prevention and control (12). Despite the existence of a variety of TBPs, few studies have focused on coinfection with multiple pathogens (13), and this is the first study investigating the coinfection of TBPs both in wild hedgehogs and their harboring ticks in China, to our knowledge.

Though various species of ticks have been found to infest *Erinaceidae* in previous studies depending on the diversity of geographical environment they inhabit (14, 15), such as *Hyalomma* spp., *Rhipicephalus* spp., *Haemaphysalis* spp., *Ixodes* spp., et al., in this study in the Jiangsu province of Eastern China, the predominant ticks infesting *Er. amurensis* were *H. flava* (96.7%). This is consistent with other studies conducted in Jiangxi province, Eastern China, and Hubei province, Central China, in which all the 13 and 125 ticks collected from *Erinaceidae* were *H. flava* (11, 16). These studies, together with the present study, to some extent, reflect the parasite characteristics of *Erinaceidae* in China, while more studies are needed to verify the prediction, due to the small number of studies focusing on this area.

To get a general picture of the pathogen profile of ticks, mNGS has been conducted, which is useful to investigate the microbiomes and microbial diversity of ticks (17). Jiao et al. applied mNGS to investigate TBPs in cattle-attached ticks in Northern China, and proved that various pathogens including R. raoultii, “*Candidatus* R. tarasevichiae,” Anaplasma sp. Mongolia, Coxiella-like endosymbiont (CLE), and Babesia venatorum coinhabited Dermacentor nuttalli and Ixodes persulcatus (13). Another similar study in Japan confirmed the existence of *Coxiella* spp., *Ehrlichia* spp., and *Rickettsia* spp. in salivary glands of various ticks (including H. flava) by mNGS (18). Similarly, in the present study, the presence of 4 kinds of important genera, including *Rickettsia*, *Anaplasma*, *Ehrlichia*, and *Coxiella*, were detected by mNGS in the pooled sample of ticks, with *Coxiella* the most abundant. The mNGS procedure effectively cleared the fog in front of our targets, and reduced ineffective labor, so that we could evaluate the prevalence of specific genera of pathogens in the nested PCR. In general, coupling mNGS with PCR, 9 and 4 species of TBPs were identified in *H. flava* and *Er. amurensis* in the present study, respectively.

*R. japonica* is still prevalent in China and Japan in recent years and can cause Oriental spotted fever (19 to 22). Interestingly, the H. flava ticks had a high positive rate (81.1%) of R. japonica, and their hedgehog hosts should also have a high R. japonica-positive rate. However, the reality was just the opposite, with neither the *gltA* nor the *rrs* gene of R. japonica detectable in the organs of these *Er. amurensis* hedgehogs. We supposed that R. japonica didn’t cause chronic infection and the pathogen transmitted by tick biting would be eliminated by the hedgehog's immune system quickly, based on the following three reasons. First, nowadays, the only existent Rickettsia sp. in hedgehogs reported was R. helvetica (8), which is also the only species that could establish chronic infection in SFGR (23). Second, C. burnetii, A. bovis, and Ehrlichia sp. could be detected in hedgehogs in the present study, and all of them have the ability to establish chronic infections. Third, in our previous study (24), when we did a preliminary experiment using R. heilongjiangensis, a species with a high degree of similarity with R. japonica at the genomic level, to infect healthy C3H/HeN mice, the bacterial load in mouse organs (including spleen, liver, and lung) dropped quickly since day 3 postinfection, until total elimination in days 7 to 10, and the elimination procedure of *R. japonica* might also happen in hedgehogs. Thus, very few organisms existed in the organs beyond the range of detection limit of nested PCR. In a recent study in Tunisia, similar results were observed in *Atelerix algirus* hedgehogs, who were infested with *Rickettsia*-positive ticks (positive rate 47.2%) and fleas (positive rate 79.2%), while the *gltA* and *ompB* genes of *Rickettsia* were not detected in the hedgehog organ samples by conventional or nested PCR (25).

Considering the high prevalence of R. japonica in the ticks, we hypothesize that Er. amurensis hedgehogs just play a role of “bridge” that transmits R. japonica from an infected H. flava tick to an uninfected one attached by the same host (26), but not as a reservoir host or infectious source. In this “bridge transmission pattern,” hedgehogs may acquire either a short-term systemic infection or a nonsystemic infection that is limited to the parts where the infected ticks are feeding to transmit R. japonica to uninfected H. flava ticks. Actually, the latter transmission pattern, with systemic infection in host unnecessary, has been reported in Borrelia burgdorferi transmission between ticks feeding on sheep (27). However, the hypothesis here needs further confirmation with animal model experiments considering the small number of hedgehogs used, and it is also possible that the pathogen is vertically transmitted in ticks, and the hedgehog blood meal simply allows for propagation of SFGR in ticks without being transmitted to mammals. The other sequence SFGR-1 performed highest homology with an uncultured Rickettsia sp. sequence (KC776315.1) identified in H. longicornis in Beijing (9), indicating it may be a novel Rickettsia sp. while further characterizations of its sequences and infectivity are needed.

Both A. platys and A. bovis were identified in H. flava, while only *A. bovis* was found in organs of the hedgehogs. A. bovis was predominant in ticks compared with A. platys, and this is not unexpected, because hedgehogs may play roles in the circulation of A. bovis. A. platys is a pathogen that infects platelet of dogs and could also cause human infections (28 to 30), while *A. bovis* causes anaplasmosis in animals including bovines, raccoons, cats, deer, etc. (31 to 34). To our knowledge, this is the first time *A. bovis* has been identified in hedgehogs.

Three kinds of Ehrlichia spp. were identified in ticks and only one kind (Ehr-1) was detected in organs of Er. amurensis. In phylogenetic analysis, Ehr-1 and Ehr-2 clustered together with uncultured or undefined Ehrlichia spp., indicating they were novel Ehrlichia species and genetic diversity existed in this area. Also, they are taxonomically related to E. ewingii by sharing the same clade in the phylogenetic tree. E. ewingii has been proved to cause human granulocytic ehrlichiosis (HGE) (35), and Ehr-1 in this study was proved to infect hedgehogs. So, whether the newly identified Ehrlichia spp. could infect humans still needs further confirmation. Moreover, Ehr-1 was predominant in both ticks and hedgehogs, indicating mutual communications existed between them, and hedgehogs might play some roles in the *Ehrlichia* circulation.

Interestingly, the phenomenon observed in Coxiella spp. is the opposite of that observed in Anaplasma spp. or Ehrlichia spp. Here, the predominant Cox-1 in H. flava ticks was not identified in *Er. amurensis* hedgehogs, while the predominant C. burnetii strain (Cox-2) detected in hedgehogs had a low positive rate in ticks. This indicates Cox-1 may not have the ability to infect hedgehogs and is most likely a symbiotic bacterium, considering it shared the same clade with *Coxiella*-like endosymbiont strain CRt (CP024961.1) in the phylogenic tree (Fig. 6B). Cox-1 has a positive rate of 61.1% in ticks, which is also consistent with the typically high infection frequencies of CLE in infected tick populations (36 to 39). These bacteria act as obligate nutrient providers for ticks by encoding pathways needed for essential amino acids, major B vitamins, and cofactors synthesis (40 [Bibr B41][Bibr B42][Bibr B43]to 44). The phenomenon that C. burnetii performed at a high positive rate of 80% in hedgehogs, while a low positive rate of 10.9% in parasitic ticks indicates the horizontal transmission of C. burnetii between the hedgehogs and their infesting ticks was inhibited. Coinfected TBPs can work synergistically, indifferently, or antagonistically within their host (45). The statistical analysis on possible interactions between these identified pathogens verified that the inhibition effect on C. burnetii infection in H. flava was provided by the CLE (Cox-1), with a very low *P* value. The presence of endosymbiont species has been reported to exclude R. rickettsii as a pathogen in ticks in rickettsial interactions (45 to 48), while the similar phenomenon in *Coxiella* spp. is the first time it was observed in the world, suggesting the possibility of biological intervention to reduce the prevalence of C. burnetii among H. flava hosts. Moreover, it is also the first time it was observed that the existence of CLE is able to facilitate the coinfection of *Rickettsia* spp., though with a relatively bigger *P* value (0.1 < *P < *0.5) in the statistical analysis. Nowadays, there is still too little research on CLE, and the mechanisms behind the phenomenon need deep digging. CLE was previously detected in pools of ticks containing H. longicornis, H. flava, Rh. sanguineus, and Rh. turanicus in China, which actually could not confirm its presence in H. flava. So, according to our knowledge, this is the first identification of CLE in H. flava.

C. burnetii, the causative agent of the worldwide zoonosis, Q fever, is also a biological warfare agent (49). Ticks are the principal vector and reservoir of C. burnetii (49), and *H. flava* was reported to harbor C. burnetii previously (11). The high positive rate of C. burnetii in hedgehogs (80%) suggests *Er. amurensis* may be a potential reservoir host of this pathogen and contribute to its circulation. Moreover, the contact with *Er. amurensis* or its feces in this area may cause Q fever for humans. However, as the *rrs* gene is highly conserved, further investigations are needed to genotype the strain and confirm its virulence.

Coinfections of multiple pathogen/endosymbiont were observed to be in the majority in both ticks and their hosts in the present study, which may be a result of mutual infections between parasites and hosts during blood feeding. Similar observations on coinfections were reported in ticks collected from bovines in north China (13) and in ticks, fleas, and their hedgehog hosts in Tunisia (25). The results suggest the potential roles of hedgehogs and their parasites as simultaneous wild reservoirs and melting pots for TBPs, which may impose a threat to humans. Wild hedgehogs have become common in suburban and urban areas, searching for food and shelter. People are becoming more and more likely to be exposed to zoonotic pathogens carried by hedgehogs and their parasites. Therefore, future studies on the prevalence of coinfection with TBPs in humans living near their natural habitat are needed.

One of the limitations of our study is that we cannot conclude whether the presence of different TBPs can be entirely attributed to the ticks and their pathogenic contents, or they may be influenced by previous blood meal hosts, which is also the problem met by studies using engorged ticks (45). The coexistence of A. bovis and A. platys or Cox-1 and C. burnetii in the few ticks may be due to the host blood that the ticks fed on.

In conclusion, in the present study, we find the following. (i) *Er. amurensis* hedgehogs seem to contribute to the spread, transmission, and enzootic cycles of *R. japonica*, *A. bovis*, *Ehrlichia* sp., and C. burnetii, and they may be the reservoirs of *A. bovis*, *Ehrlichia* sp., and C. burnetii but not *R. japonica*, in which *A. bovis* is proved to infect hedgehogs for the first time in the world. (ii) *H. flava* is proved to harbor various TBPs as a reservoir host, including CLE identified for the first time, and the existence of CLE may inhibit coinfection of C. burnetii but facilitate that of SFGR in *H. flava.* (iii) Four novel TBP species were identified, including one *Rickettsia* sp., two *Ehrlichia* spp., and one CLE. Though some hypotheses in this study still need further investigations and confirmations, the prevalence and coinfection data of various important TBPs in *Er. amurensis* hedgehogs and their parasites *H. flava* provide useful information and data for evaluating the potential risk of human infections, thus benefiting the early warning, prevention, and control of potential diseases. Moreover, the more we learn, the more we want to know, including whether hedgehogs can maintain these pathogens in natural cycles independently, their contributions to the TBPs’ enzootic cycle, and the transmission mechanisms involving hedgehogs and ticks, all of which remain to be investigated.

## MATERIALS AND METHODS

### Hedgehogs and ticks.

Thirty hedgehogs were collected in the town of Tianquanhu (E 118°35′20″, N 32°47′20″), Huaian city, Jiangsu province, China during 2016 to 2018 (Fig. S1). All of them died from car accidents or some other unknown reasons and were donated by the local villagers. These hedgehogs died not long (within 48 h) before we got them. After the ticks were collected, the hedgehogs were dissected for collection of various organs including spleens, livers, lungs, intestines, brains, hearts, and kidneys.

All the 181 adult ticks collected were feeding on the hedgehogs. In a simple random sampling method, 4 to 10 ticks for each animal were collected and initially identified by morphological features, followed by molecular methods described previously (50).

Animal samples were used only with the approval of the Ethics Committee at Huadong Research Institute for Medicine and Biotechniques.

### DNA purification.

Ticks that had been stored in ethanol were thoroughly washed twice with phosphate-buffered saline (PBS) before being homogenized individually in 1 mL of PBS. An appropriate amount of each homogenate or organ sample was used to extract their DNA using a QIAamp Fast DNA Tissue kit (Qiagen, Dusseldorf, Germany) in accordance with the manufacturer's instructions and stored at −20°C prior to analysis.

### Metagenome next-generation sequencing and data analysis.

A pooled DNA sample was prepared by mixing 20 μL of individual DNA extractions from 10 randomly selected *H. flava* ticks for complete microbial genome sequencing using mNGS as described previously (13). Sequencing was conducted using the Illumina HiSeq platform by Sangon Biotech company (Shanghai, China). For quality control, the obtained raw data were trimmed and validated using Trimmomatic software to get clean reads (51). Raw data were filtered to remove reads with more than 10 “N” bases. Low-quality bases (quality value <20) on both ends as well as the adapters on the reads were removed. The clean reads were assembled to contigs using IDBA-UD software based on the De Bruijn graph mechanism (52). A Prodigal program was used to predict the open reading frames of the contigs (≥100 bp) and translate them into amino acid sequences (53). CD-HIT (V4.5.8) was used to establish a nonredundant gene catalog, and Bowtie2 was used to align the clean reads (54). The number of successfully aligned reads was calculated using Samtools (55), and based on the number and the length of the genes, the relative abundance of genes was calculated as previously described (13). The obtained amino acid sequences were aligned with the NCBI NR (NCBI nonredundant protein sequences) database using BLAST software to obtain functional annotations and homologous species information. Meanwhile, according to the NCBI microbial taxonomic information database, the annotation information of species classification of genes was obtained, and each taxonomic level was evaluated, including the kingdom, phylum, class, order, family, and genus.

### PCR and sequencing.

The presence of TBPs identified by mNGS was further confirmed by genus-/group-specific nested PCR targeting *rrs* genes of *Anaplasma* spp., *Ehrlichia* spp., *Coxiella* spp., and spotted fever group *Rickettsia* (SFGR) in individual *H. flava* ticks and hedgehogs as described previously (11, 13). For SFGR, primers for the *gltA* gene were also used to detect samples from hedgehog organs. For *Anaplasma* spp. and *Ehrlichia* spp., first, a universal nested PCR primer set resulting in a short partial sequence of the *rrs* gene around 280 bp was used as described previously (13), and then by nested PCR, a longer partial *rrs* gene and *gltA* gene were amplified for *Anaplasma*- and *Ehrlichia*-positive samples, respectively.

Species of each hedgehog and tick was identified by PCR amplification of corresponding partial mitochondrial 16S rRNA gene fragment as described previously (50, 56), and the amplification procedure for ticks was also used as a quality control to ensure the successful extraction of DNA.

In ordinary PCR and the nested PCR, 1 μL of the extracted DNA sample was used as the template. Premix *Taq* kit (TaKaRa, Beijing, China) was used to carry out the PCR amplifications according to the manufacturer’s instructions. Negative and positive controls were conducted using ddH_2_O and DNA of corresponding pathogens, respectively. Amplified products were analyzed by agarose gel electrophoresis, and then sequenced by the Sangon Biotech company (Shanghai, China). The primers used are indicated in Table 2.

**TABLE 2:** PCR primers used in this study

Object species	Target genes	Primer names	Sequences (5′–3′)	Round
Spotted fever group *Rickettsia*	*rrs*	S1	TGATCCTGGCTCAGAACGAAC	1st
		S2	TAAGGAGGTAATCCAGCCGC	1st
		S3	AACACATGCAAGTCGRACGG	2nd
		S4	GGCTGCCTCTTGCGTTAGCT	2nd
	*gltA*	CS2d	ATGACCAATGAAAATAATAAT	1st
		CSEndr	CTTATACTCTCTATGTACA	1st
		RpCS.877p	GGGGACCTGCTCACGGCGG	2nd
		RpCS.1258n	ATTGCAAAAAGTACAGTGAACA	2nd
*Anaplasma* spp. and *Ehrlichia* spp.	*rrs*	Eh-out1	TTGAGAGTTTGATCCTGGCTCAGAACG	1st
		Eh-out2	CACCTCTACACTAGGAATTCCGCTATC	1st
		Eh-gs1	GTAATAACTGTATAATCCCTG	2nd
		Eh-gs2	GTACCGTCATTATCTTCCCTA	2nd
*Anaplasma* spp.	*rrs*	An16s1	GTCACTGACCCAACCTTAAATGGCTGC	1st
		An16s2	ATCCTGGCTCAGAACGAACGCTGG	1st
		An16s3	GCGCCCTTCCGTTAAGAAGGATCTA	2nd
		An16s4	AGCTTAACACATGCAAGTCGAACGGA	2nd
*Ehrlichia* spp.	*gltA*	e-gltAwf	TTCTCAGGAATACATGCCACC	1st
		e-gltAwr	ACCATTGAGCAGACCAGCCA	1st
		e-gltAnf	AATTGCAGGGATAGTGGCAA	2nd
		e-gltAnr	CTGTGGCCAAAACCCATCAA	2nd
*Coxiella* spp.	*rrs*	Cox16SF1	CGTAGGAATCTACCTTRTAGWGG	1st
		Cox16SR2	GCCTACCCGCTTCTGGTACAATT	1st
		Cox16SF1	CGTAGGAATCTACCTTRTAGWGG	2nd
		Cox16SR1	ACTYYCCAACAGCTAGTTCTCA	2nd
Tick	Large subunit ribosomal RNA gene	TickHF	GGTATTTTGACTATACAAAGGTATTG	1st
		TickHR	TTATTACGCTGTTATCCCTAGAGTATT	1st

### Phylogenetic analysis.

SnapGene software (snapgene.com) was used to evaluate the quality of the sequencing data, and only data of high quality were used. The obtained nucleotide sequences, with primer sequences removed on both ends, were analyzed using the BLAST search engine online (https://blast.ncbi.nlm.nih.gov/Blast.cgi) in the GenBank database to obtain sequences with high homology. Corresponding sequences from whole genomes were preferentially selected when available.

Multiple sequence alignments among the obtained sequences and the selected sequences were performed using the ClustalW tool of MEGA 7.0 software, and phylogenetic analyses were performed for *rrs* of SFGR, *Coxiella* spp., and *Anaplasma* spp., and for *gltA* of *Ehrlichia* spp. using the maximum likelihood method with the number of bootstrap samples at 1,000 in MEGA 7.0 software.

### Statistical analysis.

The mutual effect that the presence of one pathogen imposed on the coinfection of another pathogen was statistically analyzed. Determined by the sample volume (*n*) and theoretical frequencies (T), the appropriate test chosen from chi-square test, continuity adjusted chi-square test, and Fisher's exact test was used as described previously (57). A *P* of* <*0.05 indicated a significant correlation between the pathogens. The promotion or inhibition effects of individual pathogens on the other pathogens were judged by their actual frequencies.

### Data availability.

The generated mNGS data have been deposited into the GenBank database under accession number PRJNA841387 and are accessible with the following link: https://www.ncbi.nlm.nih.gov/sra/PRJNA841387. The nucleotide sequences obtained in the present study were deposited into the GenBank database under accession numbers from ON016515 to ON016529.
