# Supplementary material for: Wild Hedgehogs and Their Parasitic Ticks Coinfected with Multiple Tick-Borne Pathogens in Jiangsu Province, Eastern China
Source: Microbiol Spectr. 2022 Aug 24;10(5):e02138-22. doi: 10.1128/spectrum.02138-22 (PMC9602733; doi:10.1128/spectrum.02138-22)
Supplement: Supplemental file 1 — Supplemental material. Download spectrum.02138-22-s0001.pdf, PDF file, 0.4 MB [file spectrum.02138-22-s0001.pdf]

Supplementary Figures

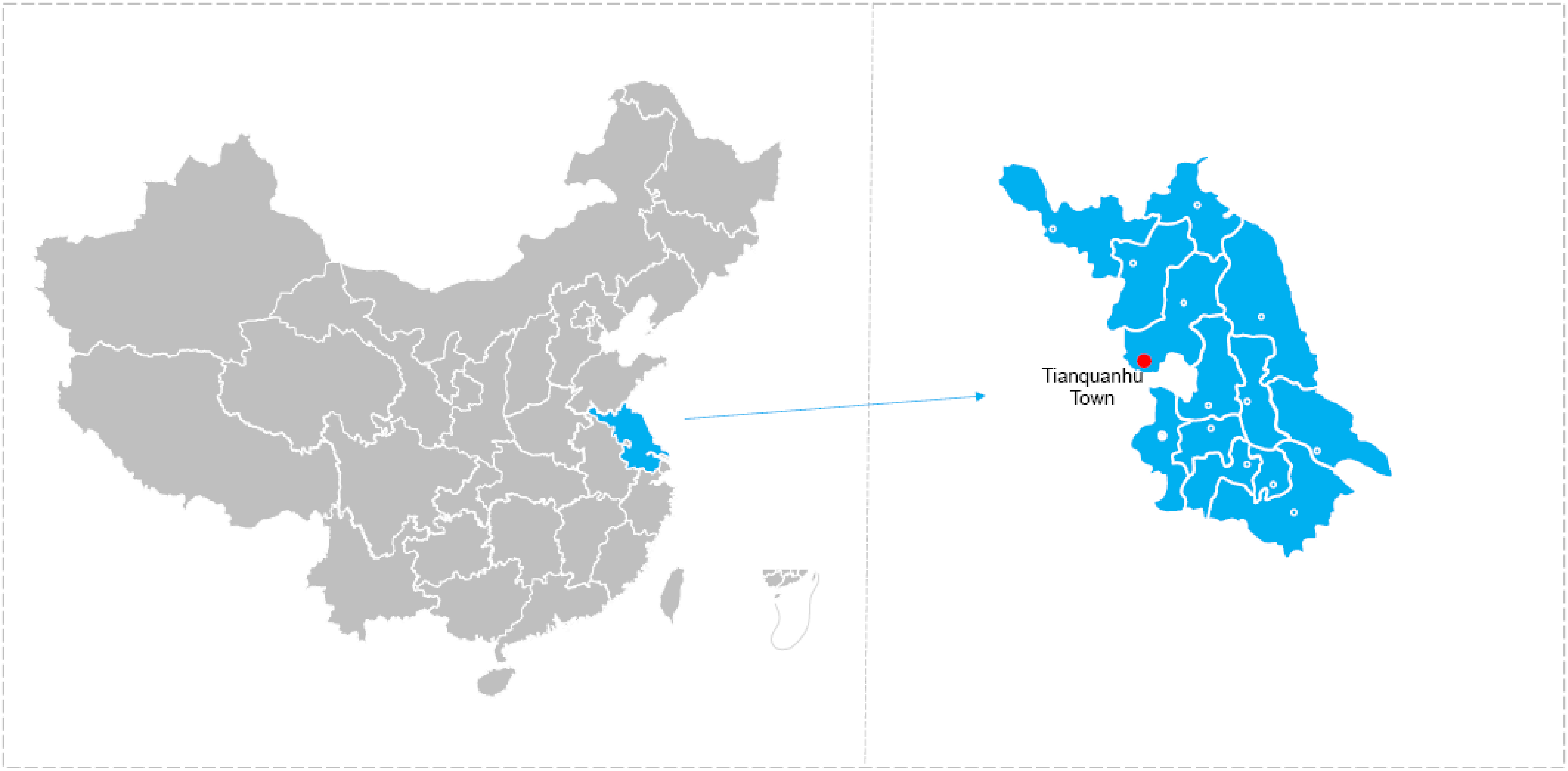

Supplementary Figure 1 Sampling location of this study.

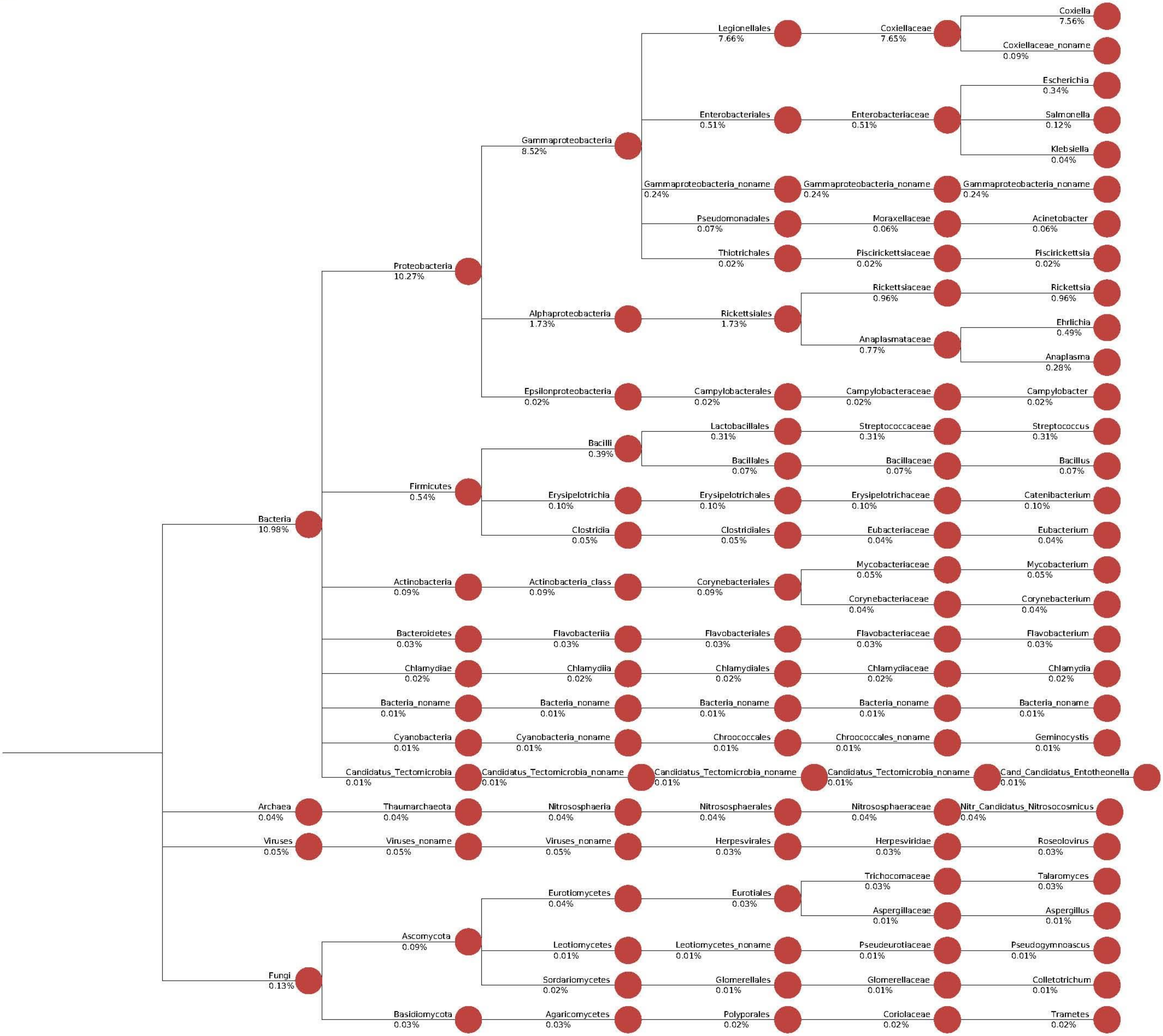

Supplementary Figure 2 Relative abundance at taxonomic levels of kingdom, phylum, class, order, family, and genus.

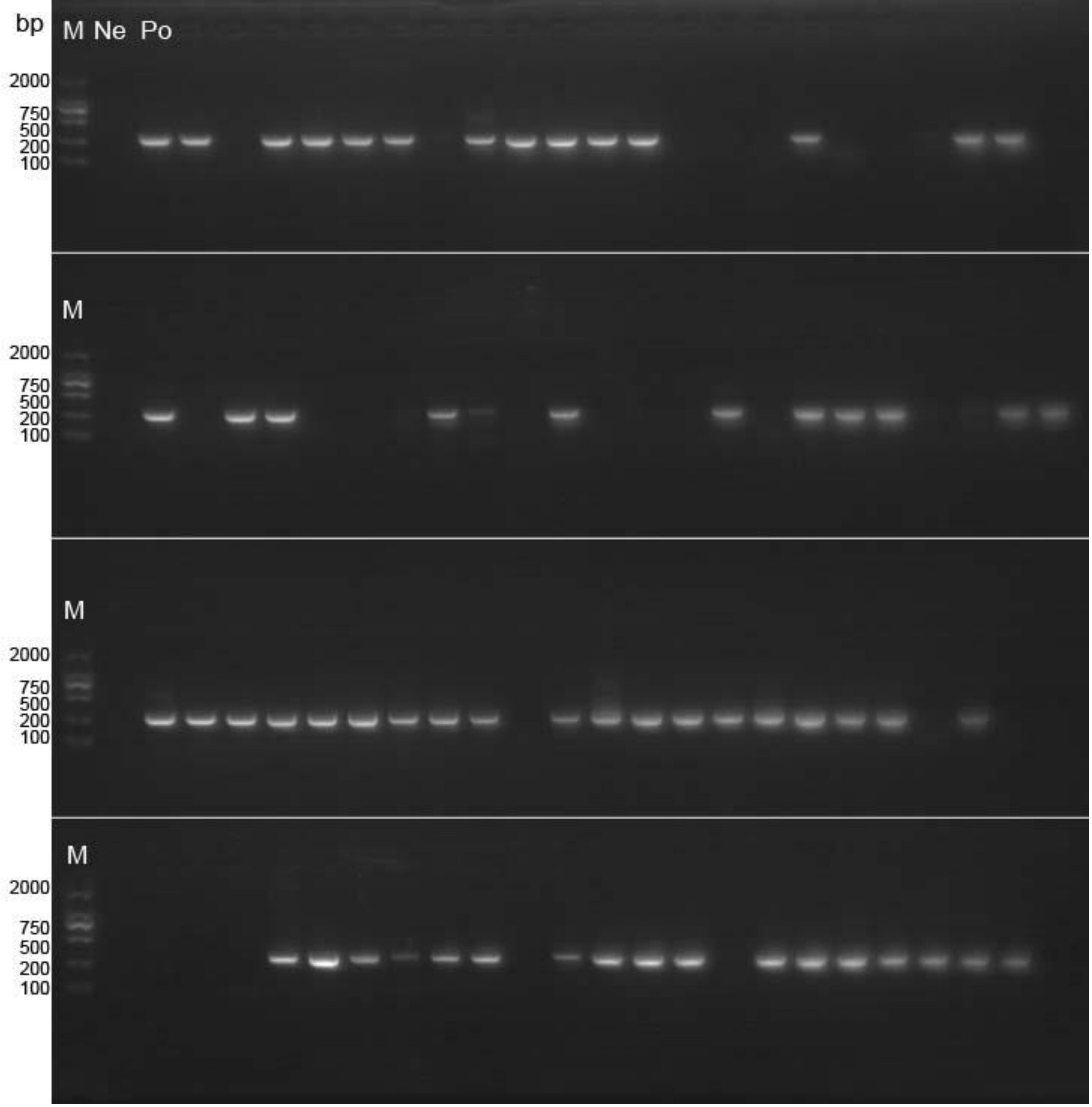

Supplementary Figure 3 The representative results of agarose gel electrophoresis analysis of the amplified products of nested PCR towards partial *rrs* genes of *Anaplasma* spp. and *Ehrlichia* spp. in hedgehog organ samples.
